# Loss-of-function variants in *SAXO6*, encoding a microtubule inner protein of photoreceptor cilia, cause a late-onset retinal dystrophy

**DOI:** 10.1016/j.ajhg.2026.02.001

**Published:** 2026-02-24

**Authors:** Abigail R. Moye, Caitlyn L. McCafferty, Siying Lin, Ji Hoon Han, Lubica Dudakova, Kim Rodenburg, Viktória Szabó, Zoltán Zsolt Nagy, Dinah Zur, Marie Vajter, Bohdan Kousal, Alexandre P. Moulin, Alexandra Graff-Meyer, Susanne Roosing, Omar A. Mahroo, Gavin Arno, Andrew R. Webster, Tamar Ben-Yosef, Petra Liskova, Benjamin D. Engel, Ditta Zobor, Mathieu Quinodoz, Carlo Rivolta

**Affiliations:** 1 Institute of Molecular and Clinical Ophthalmology Basel (IOB), 4031 Basel, Switzerland; 2 Department of Ophthalmology, University of Basel, 4031 Basel, Switzerland; 3 Biozentrum, University of Basel, Spitalstrasse 41, 4056 Basel, Switzerland; 4 Division of Evolution, Infection and Genomics, School of Biological Sciences, Faculty of Biology, Medicine and Health, University of Manchester, Manchester M13 9NT, UK; 5 Manchester Centre for Genomic Medicine & Department of Ophthalmology, Saint Mary’s Hospital & Manchester Royal Eye Hospital, Manchester University NHS Foundation Trust, Manchester M13 9WL, UK; 6 National Institute of Health Research Biomedical Research Centre at Moorfields Eye Hospital and the UCL Institute of Ophthalmology, London EC1V 2PD, UK; 7 UCL Institute of Ophthalmology, University College London, London EC1V 9EL, UK; 8 Department of Pediatrics and Inherited Metabolic Disorders, First Faculty of Medicine, Charles University and General University Hospital in Prague, 128 08 Prague, Czech Republic; 9 Department of Human Genetics, Radboud University Medical Center, 6525 GA, Nijmegen, the Netherlands; 10 Department of Ophthalmology, Semmelweis University, 1085 Budapest, Hungary; 11 Ophthalmology Division, Tel Aviv Sourasky Medical Center, affiliated to Faculty of Medical & Health Sciences, Tel Aviv University, Tel Aviv-Yafo 6997801, Israel; 12 Department of Ophthalmology, First Faculty of Medicine, Charles University and General University Hospital in Prague, 128 08 Prague, Czech Republic; 13 Jules-Gonin Eye Hospital, Fondation Asile des Aveugles, University of Lausanne, 1004 Lausanne, Switzerland; 14 Friedrich Miescher Institute for Biomedical Research, Basel, Switzerland; 15 Division of Research, Greenwood Genetic Center, Greenwood, SC 29646, USA; 16 The Ruth & Bruce Rappaport Faculty of Medicine, Technion-Israel Institute of Technology, Haifa 31096, Israel; 17 Department of Genetics and Genome Biology, University of Leicester, Leicester LE1 7RH, UK

**Keywords:** inherited retinal diseases, retina, microtubule-associated protein, cilia, ciliopathy, retinitis pigmentosa, MDM1, SAXO, microtubule inner protein, cross-linking/mass spectrometry

## Abstract

Over 500 genes have been linked to various forms of inherited retinal diseases (IRDs), a class of Mendelian conditions that affect the survival and function of rod and cone photoreceptors and, in most instances, lead to progressive visual loss. Yet some affected individuals still lack a clear genetic diagnosis, suggesting that more disease-associated genes remain to be discovered. Following the genetic analysis of extended cohorts of individuals diagnosed with late-onset recessive retinal dystrophy, we identified bi-allelic combinations of six predicted null variants in *MDM1* (now renamed *SAXO6*, stabilizer of axonemal microtubules 6) in six subjects from five families. Iterative ultrastructure expansion microscopy coupled with immuno-gold transmission electron microscopy revealed co-localization of SAXO6 with distinct ciliary microtubules from the immotile cilium present in rod and cone photoreceptors in human retina, as well as from the motile cilia present in lung epithelial cells. Cross-linking mass spectrometry uncovered an interaction between SAXO6 and α-tubulin, supporting its classification as a microtubule inner protein (MIP). These results link SAXO proteins to Mendelian conditions, highlighting the fundamental role for MIPs in the preservation of long-term retinal function.

## Graphical abstract

**Figure:**
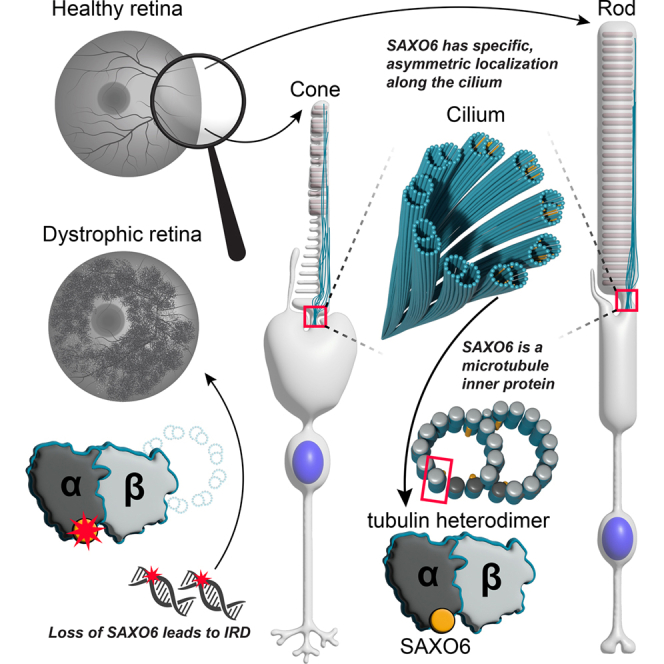

## Teaser

Photoreceptors are neurons in the retina that are responsible for detecting light. We identified pathogenic DNA variants in *SAXO6* in individuals with progressive blindness and discovered that SAXO6 is one of the proteins that likely stabilizes the distal part of photoreceptors, keeping retinal cells functional and healthy.

## Introduction

Cilia are microtubule-based organelles essential for numerous cellular processes. They are generally categorized into two types: motile cilia, which generate propulsion or fluid flow, and non-motile (primary) cilia, which serve as cellular sensors for mechanical, osmotic, and molecular signals.^1^ Every cilium contains a unique composition of lipids and proteins (i.e., receptors, ion channels, and microtubule-associated structural proteins) that allow for highly regulated, complex, and constant bidirectional trafficking of multiple substrates.^2^^,^^3^^,^^4^ Rod and cone photoreceptor neurons in the retina utilize a highly modified primary cilium that is made of both conserved ciliary structures and photoreceptor-specific unique features.^5^^,^^6^^,^^7^ Specifically, the photoreceptor cilium contains the light-sensing outer segment (OS) upon which membranous discs are anchored and where phototransduction (conversion of light into an electrical signal) occurs, as well as the connecting cilium (CC), which serves as the conduit between the biosynthetic inner segment (IS) and the OS.^8^ In the OS, the main function of the ciliary axoneme is to support discs; however, its function in the CC is similar to the transition zone (TZ) observed in other primary cilia, acting as a ciliary gate and trafficking hub. In murine rods, the CC is approximately 1,100 nm long and 300 nm in diameter, displaying conserved structural features such as Y-links, the ciliary necklace, and the inner scaffold, all of which are hypothesized to play important roles in its gating functions.^9^^,^^10^^,^^11^

Some proteins and structures within the CC display distinct spatial distributions compared to the TZ in other cilia. For example, CEP290 (centrosomal protein 290) is confined to the TZ base in most cilia, while in rods it is distributed throughout the CC.^9^ In addition to these well-known and often studied CC proteins, there are other groups of proteins, microtubule inner proteins (MIPs) and microtubule-associated proteins (MAPs), that help maintain ciliary integrity, influence ciliary trafficking and length, and have also been shown to play important roles in photoreceptor cilium homeostasis (reviewed in Bodakuntla et al.,^12^ Gui and Orbach,^13^ and Ichikawa and Bui^14^). While the functions of a wide array of MAPs have been extensively studied in cellular and ciliary microtubules, the roles of ciliary MIPs are less well defined. Their functions are hypothesized to be specialized scaffold proteins, stabilizing proteins, or regulators of intraflagellar transport along ciliary axonemes.^15^^,^^16^^,^^17^ Though there have been many MIPs identified and mapped in cilia from different species,^18^^,^^19^^,^^20^^,^^21^^,^^22^ their location in photoreceptor cilia has not been confirmed.

Ciliopathies are inherited genetic diseases caused by variants in ciliary genes and affecting multiple tissues and organs, including the retina, kidneys, brain, and liver.^23^^,^^24^ In some instances, the retina is the sole tissue affected, resulting in conditions termed non-syndromic retinal ciliopathies,^25^ and present as a form of inherited retinal disease (IRD). Two prevalent subtypes of IRDs are retinitis pigmentosa (RP [MIM: 268000]), in which night blindness is followed by loss of diurnal peripheral and then central vision,^26^ and cone-rod dystrophy (CRD),^27^^,^^28^ in which cone photoreceptor defects lead to decreased central visual acuity and photophobia. There are more than 500 genes associated with IRDs, with between 17% and 25% of them shown to be cilia-related genes.^29^^,^^30^ This vulnerability of the photoreceptor cilium to genetic defects underscores the critical role of cilia in retinal health and function. In addition, despite the development of increasingly powerful sequencing technologies such as short-read whole-exome sequencing (WES), short-read whole-genome sequencing (WGS), and long-read sequencing,^31^ as well as bioinformatic tools to analyze these large datasets,^32^^,^^33^ the genetic diagnostic rate remains only between 50% and 80%.^31^^,^^34^^,^^35^^,^^36^^,^^37^^,^^38^ The missing heritability is often attributed to challenges in molecular genetics investigations, including prioritization of types of variants,^39^ but may also suggest that there are pathogenic variants in yet-to-be-identified IRD-associated genes.

Mouse double minute 1 nuclear protein (MDM1) is a centrosomal protein shown to interact with microtubules and suppress centriole duplication.^40^ MDM1 is also predicted to interact (through bait-prey^41^ and AlphaFold-multimer predictions^42^) with the centrosomal inner scaffold proteins POC1A and POC1B.^42^ Recently, domain mapping investigations have identified MDM1 as a candidate ciliary MIP, suggesting interactions with microtubules through its conserved Mn motifs.^43^ Murine models with loss of function of *Mdm1*, including a spontaneous mutant and an engineered knockout animal, exhibit only retinal degeneration.^44^^,^^45^ However, variants in *MDM1* (MIM: 613813) are not known to cause disease in humans.

In this study, we identified five families with variants in *MDM1* associated with both RP and CRD and revealed a co-localization between MDM1 and ciliary microtubules in human rod and cone photoreceptor cells. Furthermore, we demonstrated in motile cilia that the Mn motifs within MDM1 interact with α-tubulin (TUBA), placing it within the inner lumen of ciliary microtubules in a similar manner to other stabilizers of axonemal microtubules (SAXO) MIPs. Our data, in combination with previous studies on MDM1, create the possibility that this protein is a ciliary MIP, prompting the HUGO Gene Nomenclature Committee (HGNC) to rename this gene from *MDM1* to *SAXO6*. Altogether, this work establishes a clear association between MIPs and IRDs, linking human disorders to SAXO protein dysfunction.

## Subjects, material, and methods

### Clinical assessment

This study adhered to the tenets of the Declaration of Helsinki and was approved by the ethics committees of respective institutions (Ethikkommission Nordwest- und Zentralschweiz, the London - Camden & Kings Cross Research Ethics Committee, Wales REC5, the North West of England Research Ethics Committee, the Ethics Committee of Tel Aviv Sourasky Medical Center, the Ethics Committee of the General University Hospital in Prague, and the Ethics Committee of the Medical Research Council Hungary). Written informed consent was obtained from all individuals or their legal guardians prior to their inclusion in this study.

All patients underwent a complete and standardized ophthalmic evaluation, including assessment of the best corrected visual acuity (BCVA) converted to decimal values, slit-lamp evaluation, fundoscopy, and visual field testing (Goldmann kinetic perimetry or static automated perimetry). Color fundus photography and fundus autofluorescence (FAF) imaging were acquired with the Clarus 700, FF 450 plus IR (Carl Zeiss Meditec AG, Jena, Germany), or Optos ultrawide-field (UWF) pseudocolor imaging (Optos 200Tx, Optos, Dunfermline, UK). Spectral domain optical coherence tomography (SD-OCT) to obtain macular scans was performed with Spectralis (Heidelberg Engineering, Heidelberg, Germany). Electroretinograms were performed incorporating the International Society for Clinical Electrophysiology of Vision (ISCEV)^46^^,^^47^ using either gold foil corneal recording electrodes or Burian-Allen or ERG-Jet contact lens electrodes (Hansen Ophthalmic Laboratories, Iowa City, IA, USA) and the E3 Espion (Diagnosys, Lowell, MA, USA) or the RETIport/scan 21 system (Roland Consult, Brandenburg, Germany). The patients’ electroretinography (ERG) amplitudes were compared with controls recorded by each different ERG system and laboratory.

### DNA sequencing and data processing

DNA was obtained from blood or saliva samples by standard methods and was used as a template for WES or WGS.

Members of families I–V were analyzed by WES, as previously described.^37^^,^^48^^,^^49^ Briefly, the processing of the sequencing data (mapping, variant calling, and variant annotation) was performed by using BWA mem (v.0.7.17),^50^ Picard (v.2.14.0-SNAPSHOT) (http://broadinstitute.github.io/picard), and GATK (v.4.1.4.1)^51^ for mapping to the human genome reference sequence (build hg19/GRCh37) and variant calling. For variant annotation, we used ANNOVAR,^52^ including dbNFSP v.4.7a,^53^ with the addition of deleteriousness prediction from MutScore^54^ and splicing predictions by MaxEntScan^55^ and SpliceAI.^56^ After a first analysis focusing on genes known to be associated with IRDs, we investigated coding variants or variants with predicted impact on splicing in all protein-coding genes. The proband of family III was further analyzed by WGS, according to methods reported previously.^57^ In families IV and V, affected individuals initially underwent clinical WGS, which did not identify a pathogenic genotype in known IRD-associated genes.

All variants were validated using VariantValidator,^58^ described in accordance with the Human Genome Variation Society (HGVS) nomenclature,^59^ and classified using ACMG criteria and ClinGen recommendations (https://clinicalgenome.org/tools/clingen-variant-classification-guidance/).^60^^,^^61^ Validation of next-generation sequencing (NGS) data and intrafamilial segregation analysis were performed by DNA Sanger sequencing on PCR products, according to standard protocols.^62^ PCR conditions are available upon request. Primers used are listed in Table S1.

### Long-read RNA sequencing

We downloaded BAM files from the work by Riepe et al.^63^ on three retina samples (IDs: ccs, m64167e_210819_012015_ccs, and m64167e_210902_121011_ccs). We first created FASTQ files using BEDTools bamtofastq.^64^ The reads were then mapped to the reference genome (hg38) using minimap2^65^ (options used were as follows: -ax splice -uf --secondary = no -C5 -O6,24 -B4). The expression levels of isoforms were then computed using isoquant^66^ (options used were as follows: --complete_genedb --fl_data --sqanti_output --count_exons --model_construction_strategy fl_pacbio --check_canonical --transcript_quantification unique_only --gene_quantification unique_only --splice_correction_strategy default_pacbio --data_type pacbio_ccs).

### Immunofluorescence

For immunofluorescence staining, 5 μm human retinal formalin-fixed paraffin-embedded (FFPE) sections were deparaffinized in Neo-Clear (Sigma, Burlington, MA, USA) and then rehydrated in increasing dilutions of ethanol:water. Antigen retrieval was performed by heating the slides at 110°C for 20 min in 0.01 M citrate buffer (supplemented with 0.05% Tween 20) at pH 6. After cooling, the sections were permeabilized in 0.2% fish skin gelatin + 0.25% Triton X-100 in 1× phosphate-buffered saline (PBS) for 10 min. Blocking for 1 h in permeabilization buffer + 5% bovine serum albumin preceded primary antibody incubation overnight at 4°C. After washing in 1× PBS 3 times for 5 min, slides were incubated for 1 h with secondary antibodies. Finally, after washing in 1× PBS, slides were incubated with True Black to quench autofluorescence for 1 min before being mounted in ProLong Glass Antifade Mountant (Thermo Fisher Scientific, Waltham, MA, USA) and covered with #1.5 coverslips (Thermo Fisher Scientific). Staining with the secondary antibody only was used as a negative control to demonstrate the lack of autofluorescence in the tissue.

Immunofluorescence staining was performed in lung epithelial cells (a gift from Dr. Urs Jenal, Biozentrum, Basel, Switzerland) at 33 days, which were grown in an air-liquid interface on 6.5 mm Transwell inserts (Corning, Corning, New York, USA) with a 0.4 μm pore size, according to the protocol established in Swart et al.^67^ Immunocytochemistry was performed following their established protocol.^67^ Briefly, cells were washed and permeabilized in Triton X-100 for 20 min. Following a formalin fixation, cells were immunostained with primary and secondary antibodies for 3 h each. After washing, cells were mounted in ProLong Glass Antifade Mountant (Thermo Fisher Scientific) and covered with #1.5 coverslips (Thermo Fisher Scientific).

Imaging was performed on an Olympus FV3000 scanning confocal microscope with a 60× UPLSAPO NA 1.42 oil objective (Hachioji, Tokyo, Japan).

For iterative ultrastructure expansion microscopy (iU-ExM), human retinal tissue (67-year-old donor, female, uveal melanoma, enucleation; 2-month-old donor, female, autopsy) was obtained within 1 h after cessation of circulation and fixed in 4% paraformaldehyde (PFA) at room temperature (RT) for 15 min. Anchoring, gelation, staining, and expansion were carried out as previously described^68^^,^^69^ and are outlined as a schematic in Figure S1A. The only changes pertained to antibodies/dilutions, as listed in Table S1. Lung epithelial cultured cells were fixed in 10% formalin for 15 min at RT, and expansion was performed using the exact same protocol as for the retinal tissue. After gelation, the Transwell insert detached. Antibody dilutions and solutions used were the same as for the retina.

An expansion factor of ∼10× was obtained, calculated by taking the full width half max (FWHM) of expanded axoneme widths and dividing by 186 nm (known microtubule doublet [MTD] axoneme diameter in the CC of rods and in the axoneme of motile epithelial cells). Imaging was performed on 35-mm glass-bottom dishes with a 10-mm microwell (MatTek Life Sciences, Ashland, MD, USA) coated in poly-L-lysine (Merck, Darmstadt, Germany). A gel slice was gently placed on the microwell of the dish, a drop of water was added to the slice, and it was topped with a glass coverslip to prevent drift or dehydration. The imaging was performed on a Stellaris 8 Falcon (Leica, Wetzlar, Germany) using HyD lasers and a 40× HC PL APO CORR CS2 water immersion objective (NA 1.10) with an optical zoom between 2 and 7.

### Immuno-gold transmission electron microscopy

Following human retina dissection (67-year-old donor, female, uveal melanoma, enucleation; 2-month-old donor, female, autopsy), the retinas were pre-fixed with 4% PFA in Ames’ media (Sigma) for 15 min and prepared as previously described.^69^ Briefly, following blocking (15% normal goat serum, 5% bovine serum albumin [Sigma] + 0.5% BSA-c [Aurion, VWR, Radnor, PA, USA] + 2% fish skin gelatin [Sigma] + 0.05% saponin [Thermo Fisher Scientific] + 1× protease inhibitor cocktail [GenDepot, Katy, TX, USA]) in low-adhesion microcentrifuge tubes (VWR, Radnor, PA, USA), retinas were immunolabeled for 2.5 days at 4°C in primary antibodies (Table S1) before successive rinsing in 2% normal goat serum in Ames’ media (except for the negative control, which was incubated only in block buffer; Figure S1D). Retinas were then incubated with secondary antibodies (Table S1) overnight at 4°C. After washing, retinas were post-fixed for 1 h at RT in 2.5% PFA + 2.5% glutaraldehyde + 4 mM CaCl_2_ in 0.3 M cacodylate buffer (pH 7.4). Aldehydes were quenched in 100 mM glycine (Merck) prepared in 1× PBS. Silver enhancement was performed using HQ Silver Kit (Nanoprobes, Yaphank, NY, USA) reagents in half-dram vials for 4–6 min at RT with agitation. *En bloc* staining was performed with 1% tannic acid + 0.5% glutaraldehyde in 0.1 M HEPES (pH 7.5) and 1% uranyl acetate in 0.1 M maleate buffer (pH 6.0) before ethanol dehydration and embedding in Eponate resin. 70-nm ultramicrotome sections were cut from the resin blocks using a Diatome Ultra 45° diamond knife and collected onto copper slot grids (VWR). Grids were post-stained in Uranyless (EMS Hatfield, PA, USA) for 10 min and lead citrate solution (EMS) for 10 min. Grids were imaged on a JEM-F200 cFEG microscope (JEOL, Tokyo, Japan) with a 1400 Plus electron microscope operated at 200 keV and equipped with an EMSIS XAROSA CMOS 20-megapixel camera. Radius software was used for image acquisition, and images were subsequently cropped with slight contrast adjustments in FIJI/ImageJ.^70^

### Image processing

iU-ExM images underwent Lightning processing on the Stellaris 8 Falcon (Leica) immediately following image capture. FIJI was used for image visualization and basic adjustments of all TEM and confocal imaging.

### Bovine trachea epithelial cell cilia isolation and cross-linking

Bovine tracheas were placed on ice for 10–30 min immediately after slaughter. The remainder of the preparation was performed in a 4° cold room. Tracheas were cleaned of additional tissue, and blood was rinsed out with cold PBS until the PBS ran clear. Bovine tracheas were shaken vigorously with cilia extraction buffer as previously described.^17^ Cilia were then cleaned up by successive rounds of centrifugation at 2,000 and 12,000 × *g* to remove debris. Once the sample pellet was mostly white, 8 mM disuccinimidyl sulfoxide (DSSO) in dimethyl sulfoxide (DMSO) was added to the sample and incubated at RT for 1 h, before being quenched with Tris buffer for 30 min, as described.^22^ Bovine trachea epithelial cell (BTEC) cilia were subjected to microtubule enrichment using 1% NP-40.

### BTEC mass spectrometry

Enriched BTEC axonemes were resuspended in lysis buffer (5% SDS, 10 mM TCEP, and 0.1 M TEAB) and lysed by sonication using a PIXUL Multi-Sample Sonicator (Active Motif) with the pulse set to 50, pulse repetition frequency to 1, process time to 10 min, and burst rate to 20 Hz. Lysates were incubated for 10 min at 95°C and alkylated in 20 mM iodoacetamide for 30 min at 25°C, and proteins were digested using S-Trap micro spin columns (Protifi) according to the manufacturer’s instructions. Soon after, 12% phosphoric acid was added to each sample (final concentration of phosphoric acid: 1.2%) followed by the addition of S-trap buffer (90% methanol and 100 mM TEAB [pH 7.1]) at a ratio of 6:1. Samples were mixed by vortexing and loaded onto S-trap columns by centrifugation at 4,000 × *g* for 1 min, followed by three washes with S-trap buffer. Digestion buffer (50 mM TEAB [pH 8.0]) containing sequencing-grade modified trypsin (1/25, w/w; Promega, Madison, WI) was added to the S-trap column and incubated for 1 h at 47°C. Peptides were eluted by the consecutive addition and collection by centrifugation at 4,000 × *g* for 1 min in 40 μL digestion buffer, 40 μL 0.2% formic acid, and finally 35 μL 50% acetonitrile and 0.2% formic acid. Samples were dried under vacuum and stored at −20°C until further use.

The sample was then enriched for cross-linked peptides using a GE Superdex 30 Increase 3.2/300 size-exclusion column (Cytiva). The dried peptides were resuspended in 30% acetonitrile and 0.1% TFA, and the first 12 fractions were collected and dried for mass spectrometry (MS).

Dried peptides were resuspended in 0.1% aqueous formic acid and subjected to liquid chromatography-tandem MS (LC-MS/MS) analysis using an Orbitrap Eclipse Tribrid mass spectrometer fitted with an Ultimate 3000 nano system and a FAIMS Pro interface (all Thermo Fisher Scientific) and a custom-made column heater set to 60°C. Peptides were resolved using an RP-HPLC column (75 μm × 30 cm) packed in-house with C18 resin (ReproSil-Pur C18-AQ, 1.9 μm resin; Dr. Maisch) at a flow rate of 0.3 μL/min. The following gradient was used for peptide separation: from 5% B to 13% B over 10 min to 38% B over 110 min to 95% B over 2 min followed by 18 min at 95% B and then back to 5% B over 2 min followed by 18 min at 5% B. Buffer A was 0.1% formic acid in water, and buffer B was 80% acetonitrile and 0.1% formic acid in water.

The mass spectrometer was operated in DDA mode with a cycle time of 4 s. Throughout each acquisition cycle, the FAIMS Pro interface switched between compensation voltages of −40 and −60 V with cycle times of 2 and 2 s, respectively. MS1 spectra were acquired in the Orbitrap in profile mode at a resolution of 120,000 and a scan range of 375–1,600 *m/z*, the automatic gain control (AGC) target set to “standard,” and the maximum injection time set to 50 ms. Precursors were filtered with monoisotopic peak determination set to “peptide,” the charge state was set to 4–8, a dynamic exclusion of 30 s was used, and an intensity threshold of 2e4 was used. Precursors selected for second-stage MS analysis were isolated in the quadrupole with a 1.6 *m/z* isolation window and collected for a maximum injection time of 118 ms with the normalized AGC target set to 200%, the mass range set to “normal,” and the scan range mode set to “auto.” Fragmentation was performed with stepped higher-energy collision dissociation (HCD) collision energies of 19%, 25%, and 30%, and MS2 spectra were acquired in the Orbitrap at a resolution of 60,000 in profile mode.

A cross-link search was performed using Scout^71^ against the corresponding database for each of the samples using the default values for a DSSO-KSTY search with a cross-linked spectrum match false discovery rate (FDR) cutoff of 1%.

### Statistical analysis

For width measurements, the data were tested for normality, and groups were compared using unpaired *t* tests with Welch’s correction. Graphs are displayed with the mean and standard error of the mean.

### Antibodies

The primary and secondary antibodies used are detailed in Table S1.

## Results

### Clinical examination identifies signs and symptoms of RP and CRD

**Figure 1:**
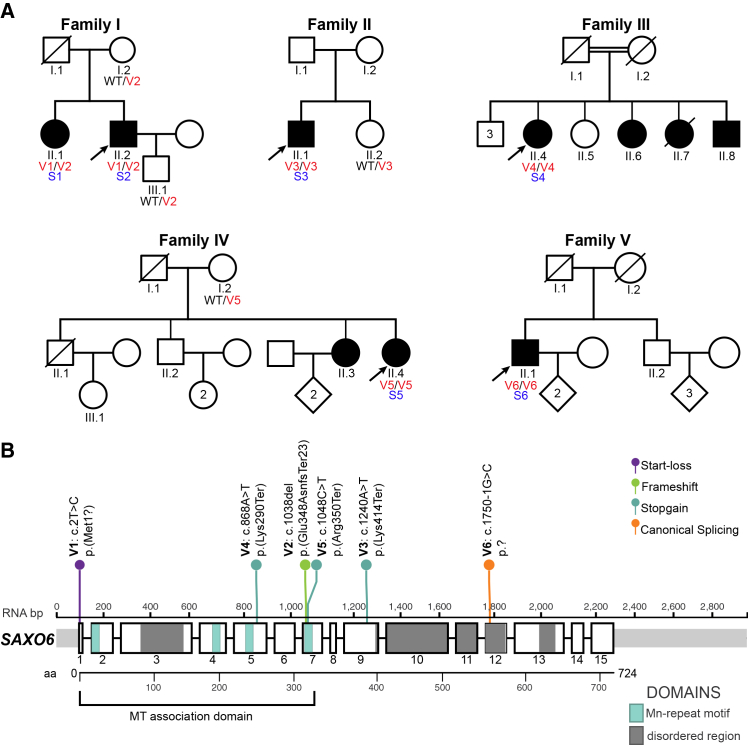
Pedigrees and genotypes of the individuals analyzed (A) Pedigrees of the families. Arrows indicate probands, and double lines indicate consanguinity. Disease-associated variants are indicated by V#; subjects are identified with S#; and “WT” represents wild-type alleles. (B) Variants detected in *SAXO6* from the subjects analyzed in this study, shown as lollipop symbols with respect to the MANE reference isoform (GenBank: NM_001354969.2 [whole transcript length: 2,980 bp, 724 aa]). The schematic is roughly proportional to the actual genome sequence, with the exons as numbered boxes and the introns as horizontal lines. Additionally, the known protein domains are highlighted, with an amino acid ruler underneath.

**Figure 2:**
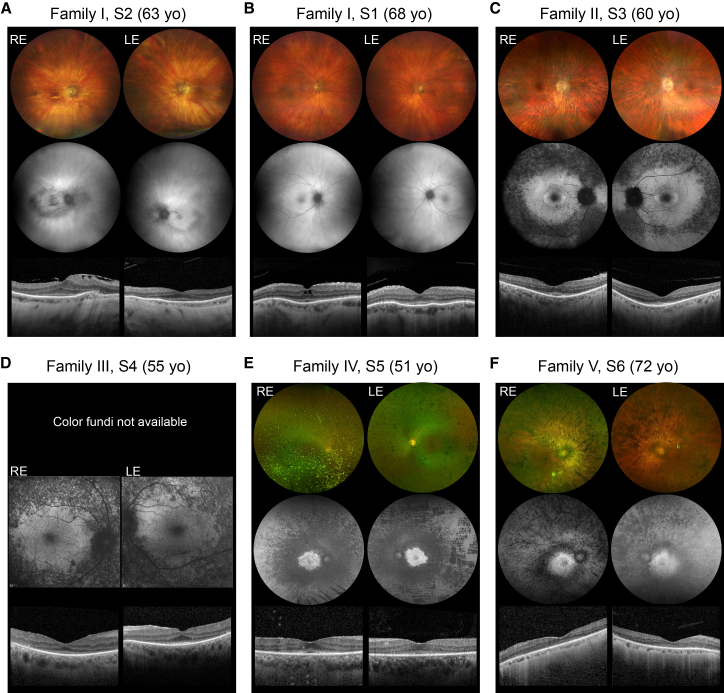
Multimodal retinal imaging (A–F) In each image, the top row shows color or pseudocolor fundus images, the middle row shows fundus autofluorescence (FAF) images, and the bottom row shows optical coherence tomography (OCT) scans. (A and B) Two individuals (S2 and S1, ages 63 and 68, respectively) with cone-rod dystrophy. (C–F) Four individuals with RP (S3, age 60; S4, age 55; S5, age 51; S6, age 72).

We ascertained six individuals with IRD from five unrelated families from the Czech Republic (family I), Hungary (family II), Israel (family III), and the UK (families IV and V) (Figure 1). All affected persons inherited the condition via a seemingly autosomal-recessive pattern. No evidence of dominant inheritance was observed. Ophthalmic examination allowed us to obtain clear diagnoses of RP (4 individuals) or CRD (2 individuals) (Figure 2; Table S2), all with visual symptoms typically emerging later in life, with a median age of onset of 44 years (minimum age: 38 years; maximum age: 67 years). Clinical details are outlined below.

The index subject from family I, S2, noticed declining vision at 43 years. At 48 years, his BCVA was 0.5 in both eyes (BEs), with better vision in dim-light conditions. The disease course and SD-OCT scans were suggestive of a cone dystrophy. At age 63, BCVA was 0.05 in BEs, corroborating a progressive deterioration in visual function over two decades. Fundus evaluation showed significant chorioretinal atrophy and attenuated blood vessels, with no retinal pigment epithelium (RPE) mottling or bone spicules. Fundus photography also documented pale optic discs and bilateral Bruch’s membrane folds (Figure 2A), which have remained unchanged since age 55. The final ocular diagnosis was CRD. His medical history included hypertension since age 40, dyslipidemia, and obesity (body mass index [BMI] = 37 when aged 63). At 62 years, he was diagnosed with bipolar disorder (MIM: 125480) and treated with oral medication.

His sister, S1, developed photophobia at 40 years, followed by reduced color perception and visual acuity. At age 68, her BCVA was 0.2 in the right eye (RE) and 0.16 in the left eye (LE). FAF distribution was irregular bilaterally, and the most significant morphological alterations were observed in the outer retina on macular SD-OCT scans (Figure 2B). Clinical ocular findings were also consistent with CRD. Her medical history indicated obesity (BMI = 40), along with hypertension and type 2 diabetes mellitus (T2DM [MIM: 125853]), which have been under management through oral medication since the age of 66 years.

Subject S3 from family II was diagnosed with RP at age 60. His night blindness began at age 45, with vision declining despite cataract surgery and yttrium aluminum garnet (YAG)-capsulotomy at 53 years. BCVA was 0.3 in the RE and 0.4 in the LE. Kinetic perimetry with target III4e showed concentric narrowing to 10° and 15° in the RE and LE, respectively. ERG recordings showed no reproducible responses (Table S2). Fundoscopy revealed pale optic discs, retinal vessel attenuation, and an intact macular area but RPE rarefication and bone spicule pigmentary changes in the mid-periphery (Figure 2C). SD-OCT showed well-maintained retinal architecture and a discernible ellipsoid zone in the fovea, with outer retinal atrophy. A hyperfluorescent macular ring and hypofluorescent mid-periphery were indicated by FAF imaging. Two years later, BCVA declined slightly to 0.3 in BEs, with stable visual fields, and FAF and SD-OCT showed minor RPE atrophy progression. At age 45, he was diagnosed with hypertension and T2DM, initially treated with oral medication and later insulin. His sister did not report any symptoms consistent with IRD.

Subject S4 from family III was diagnosed with RP at age 50. Initial BCVA was 0.05 in the RE (amblyopic) and 0.25 in the LE, with a 10° island of remaining central vision in BEs. Full-field ERG showed no reproducible responses. Optic discs were pale and waxy, with atrophic peripheral retinas and mid-periphery bone spicule deposits (Figure 2D). SD-OCT revealed preserved outer retina in the central subfoveal region with perimacular and peripheral outer retinal atrophy in BEs and epiretinal membrane in the RE. FAF identified hypofluorescent changes (Figure 2D). Over time, the LE developed cystoid macular edema, managed successfully with anti-VEGF therapy. By age 55, her vision declined to light perception in the RE and hand motion in the LE. Additionally, she was diagnosed with T2DM at age 40 and hypertension and hyperlipidemia at 55, with a normal BMI. She also has unspecified sensorineural hearing loss at age 50. In addition to herself, family III included three affected and four unaffected siblings. Two of her siblings with RP also had T2DM (the information on the third one is missing, since she is deceased), as well as two of the unaffected siblings.

Subject S5, from family IV, experienced night blindness and a reduction in her peripheral vision and was subsequently diagnosed with RP at age 38. ERGs at age 42 indicated severe retinal dysfunction, primarily affecting rods, with some inner retinal involvement in the LE and marked macular involvement. At age 51, her BCVA was 0.25 in BEs. Fundus examination revealed symmetrical retinal vessel attenuation and mid-peripheral pigment clumping (Figure 2E). FAF imaging showed mid-peripheral hypofluorescence, sparing the macula, and prior pan-retinal photocoagulation on the LE. OCT scans indicated perifoveal ellipsoid zone loss, sparing centrally. Her medical history included non-alcoholic liver cirrhosis and T2DM, diagnosed at age 38.

Subject S6 from family V, last examined at age 72, experienced a rapid deterioration in his vision beginning in his 60s, when he was first diagnosed with RP. On examination, his BCVA was light perception in BEs. Fundus examination revealed symmetrical optic disc pallor, retinal vessel attenuation, and mid-peripheral retinal atrophy with pigment clumping in BEs (Figure 2F). FAF showed mid-peripheral mottled hypofluorescence. OCT scans revealed generalized loss of outer retinal layers with no macula edema noted. His medical history included T2DM, hypertension, ischemic heart disease, monoclonal gammopathy of undetermined significance (MGUS), vitamin B12 deficiency, and iron deficiency anemia. He also developed late-onset hearing loss, for which he was fitted with hearing aids at age 71.

### Genetic analyses reveal pathogenic variants in *SAXO6*

In all ascertained affected individuals, we identified candidate pathogenic genotypes in *SAXO6*. In humans, this gene has several different isoforms, seven of which we found to be expressed in the retina through long-read RNA sequencing (Figure S2), including the canonical MANE isoform sequence GenBank: NM_001354969.2 (taken here as a reference sequence).^63^ All variants (Figure 1B; Table S3) were predicted to result in loss of functional protein, thus representing null alleles in all but two SAXO6 isoforms, and none were previously reported in ClinVar^72^ or in the literature. None of these subjects had disease-causing variants in other known IRD-associated genes.

In family I, affected siblings S1 and S2 carried two heterozygous variants: c.2T>C (p.Met1?), designated as V1, a start-loss change, and a frameshift deletion, V2 c.1038del (p.Glu348AsnfsTer23). V1 was absent from the gnomAD v.4.1 database,^73^ while V2 is very rare, as it was found only in two heterozygous individuals out of more than 800,000, resulting in an allele frequency (AF) of 1.2 × 10^−6^. Additionally, there is no alternative start codon within the vicinity of the start-loss V1 variant in the MANE isoform. Molecular analysis showed that S1 and S2 were compound heterozygotes for these variants. In family II, the affected individual S3 was homozygous for a nonsense variant, V3 c.1240A>T (p.Lys414Ter), while his unaffected sister was a heterozygote for the same variant. This DNA change was identified within a 15.7 Mb region of homozygosity that did not contain any known RP genes, and this variant in *SAXO6* was absent from the gnomAD v.4.1 database. Subject S4 from family III, a consanguineous pedigree, was homozygous for another nonsense variant, V4 (c.868 A>T [p.Lys290Ter], AF = 8.1 × 10^−6^ in gnomAD v.4.1). No additional members of this family were available for variant segregation analyses. Both individuals S5 and S6 from families IV and V, respectively, were homozygotes for predicted loss-of-function *SAXO6* variants. S5 carried the nonsense variant V5 c.1048C>T (p.Arg350Ter), present heterozygously in her mother, while S6 harbored a DNA change affecting the invariant acceptor splice site for intron 12, V6 c.1750−1G>C (p.?), predicted to result in the skipping of exon 13 and in the shift of the downstream reading frame. Co-segregation analysis could not be performed in family V, due to the unavailability of additional participants. Of note, V5 (AF = 1.2 × 10^−5^) was present in a homozygous individual from gnomAD v.4.1, who was absent from gnomAD v.2.1, most likely because the latest version of the database may contain genomes from individuals with Mendelian conditions, with the exception of severe pediatric disease.^74^ Individual S5 was also found to harbor the mitochondrial *MT-ND5* m.13063G>A (GenBank: NC_012920.1) (p.Val243Ile) variant of uncertain clinical significance at a level of ∼45% in the blood sample. This variant has only been reported once, in a 32-year-old patient with ataxia and excessive fragmentary hypnic myoclonus, where it was detected at 80% heteroplasmy in muscle and 25% in lymphocytes.^75^ The phenotype in individual S5 was not thought to be consistent with mitochondrial disease, and the contribution of this variant to their clinical phenotype remains unclear.

### SAXO6 co-localizes with individual ciliary MTDs in the cilium of human photoreceptors

**Figure 3:**
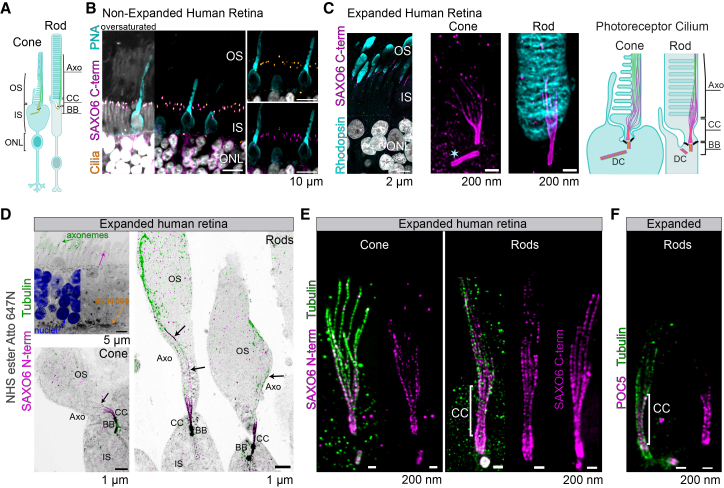
Localization of SAXO6 in human photoreceptors with iU-ExM (A) Schematic depictions of a rod and cone, including the IS, the OS, the ONL, and the synapse. Green: microtubules, orange: centrins. (B) Confocal images of formalin-fixed paraffin-embedded sections of human retina stained for centrins (orange) as a CC/BB marker, PNA (peanut agglutinin; cyan) as a cone sheath marker, and SAXO6 (magenta). (C–F) Deconvolved confocal images of expanded human retina stained with rhodopsin (cyan), tubulin (green), NHS-ester (gray), POC5 (magenta), and SAXO6 (magenta) (cones have no rhodopsin staining); the star represents the daughter centriole. A schematic representation of observed SAXO6 localization along the photoreceptor cilium is depicted in (C). (D) Different regions of photoreceptors are indicated, with black arrows pointing to axonemal SAXO6 staining. (E and F) The images of individual cilia display either SAXO6 or POC5 with tubulin on the left and further individual cilia examples without tubulin on the right. All scale bars in (C)–(F) are corrected for expansion. The CC demarcation is 1 μm (corrected for expansion). ONL, outer nuclear layer; OS, outer segment; Axo, axoneme; CC, connecting cilium; BB, basal body; IS, inner segment; M, mitochondria.

The retinal photoreceptors contain a modified primary cilium (the CC + OS) (schematic in Figure 3A). SAXO6 was previously localized to the CC and BB in mouse retina,^45^ a finding that we confirmed in human retinal FFPE sections co-immunostained for centrin (photoreceptor CC and centriole marker) and SAXO6 (Figure 3B). We used two SAXO6 antibodies, one that recognizes an epitope on the C-terminal end (labeled C-term in figures)^45^ and another that recognizes an epitope more N terminally (labeled N-term in figures) (Table S1). Because SAXO6 has been shown to directly interact with microtubules and localize to centrioles,^40^ we examined its spatial distribution in human photoreceptor cilia by using iU-ExM and immuno-gold transmission electron microscopy (Ig-TEM). iU-ExM, coupled with pan-labeling using NHS-esters (*N*-hydroxysuccinimide ester-dye conjugates interacting with amines),^76^ revealed that SAXO6 localizes to the mother basal body (BB), daughter centrioles (DCs), and throughout the CC. Most interestingly, it is present distally in the OS axoneme in both rods and cones, as shown by the extension of SAXO6 staining that overlaps with rhodopsin in the rod OS discs (Figure 3C; Video S1).

Video S1. SAXO6 in a human rod cilium with rhodopsin iU-ExM 3D z-stack of a rod cell showing SAXO6 (magenta) staining along the connecting cilium and into the outer segment, with rhodopsin (cyan) staining surrounding it. At the 00:05 time point, arrows indicate SAXO6 staining along the axial length of the rod axoneme.

When the axonemal microtubules extend into the OS, they spread apart from each other and lose their symmetrical arrangement (Figures 3D–3F).^7^^,^^69^ Likewise, SAXO6 seemed to match this pattern, protruding into the axoneme up to ∼1/3 of the observed OS length, in both rods and cones (Figures 3D and 3E; Video S2), indicating a close association with the MTDs. The localization of SAXO6 into the OS axoneme contrasts with the staining observed for many other ciliary proteins associated with IRDs, which are mostly localized to the CC or the BB only, such as POC5 (Figure 3F).

Video S2. SAXO6 in a human rod cilium iU-ExM 3D z-stack of a rod cell showing SAXO6 (magenta) staining along the connecting cilium and into the outer segment, with tubulin in green.

**Figure 4:**
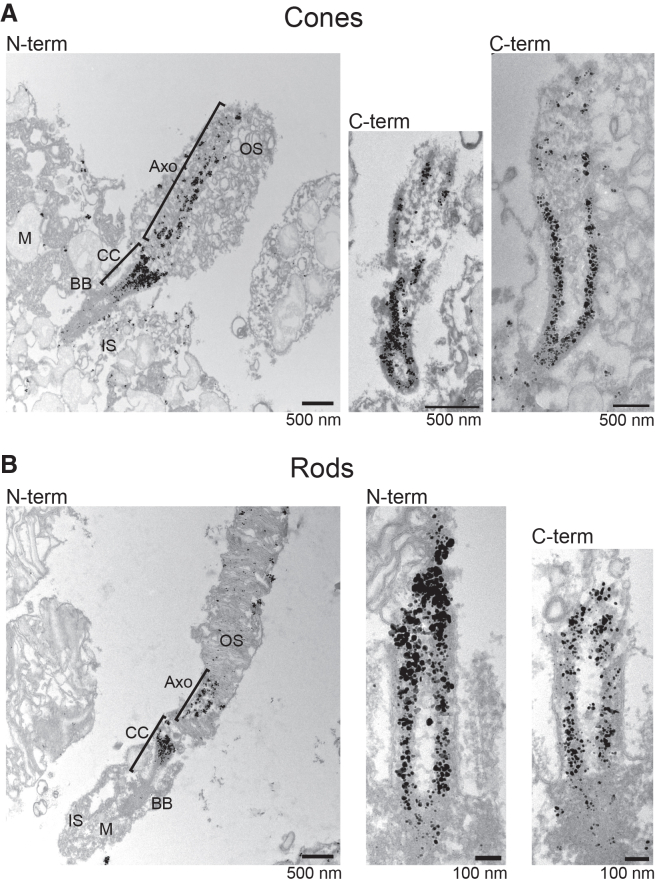
Immuno-gold localization of SAXO6 in human photoreceptors SAXO6-immuno-gold TEM (Ig-TEM) of cones (A) and rods (B). The CC demarcation is 1 μm. OS, outer segment; Axo, axoneme; CC, connecting cilium; BB, basal body; IS, inner segment; M, mitochondria.

Ig-TEM with SAXO6 in the human retina validated the findings from iU-ExM. Nanogolds labeling SAXO6 were observed in photoreceptor cilia along the CC and into the OS axoneme in both cones and rods and were observed to mostly overlap with the microtubules of the cilium instead of the ciliary membrane or luminal space (Figure 4). This contrasts with other IRD-associated ciliary proteins, such as CEP290 or RPGR, which localize to the space between the microtubules and the membrane,^69^ or POC5, which localizes within the ciliary lumen.^11^

**Figure 5:**
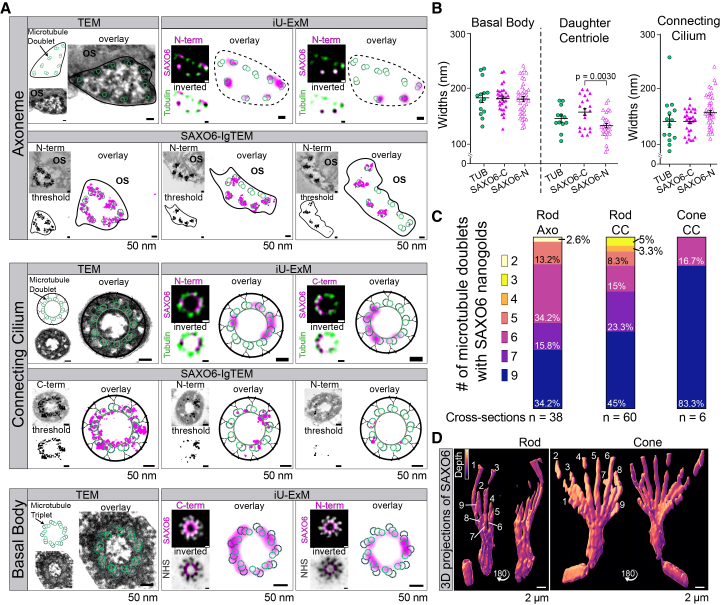
SAXO6 co-localizes with ciliary microtubules (A) Subcellular SAXO6 localization in human rods. Cross-sectional views of cilia through different levels of the photoreceptor cilium. In each image, a representative TEM micrograph of individual cilia of human rods is shown with a schematic overlay for reference. Deconvolved confocal images of individual cilia from expanded retina, stained for SAXO6 (magenta) and either tubulin (green) or NHS (gray, used for basal body structure indications), are shown with the corresponding schematic overlay. Electron micrographs of human retina immuno-gold labeled for SAXO6 were thresholded to display only the nanogold labeling and displayed with the schematic overlay. All scale bars in (A) represent 50 nm. (B) Scatterplot displaying widths of tubulin and SAXO6 labeling from individual expanded cilia in deconvolved confocal images, in either daughter centrioles, basal bodies, or connecting cilia. The mean is displayed with SEM, and *p* values were determined from unpaired *t* tests with Welch’s correction, showing a trend for decreased widths of SAXO6 N-terminal antibody labeling compared to C-terminal antibody labeling (*p* values are not shown for non-significant differences). Data were generated from four experiments, over 2 biological samples. Daughter centriole: tubulin *n* = 12, 146 + 6.42 nm; SAXO6-C *n* = 20, 157.5 ± 6.45; SAXO6-N *n* = 30, 133.1 ± 4.0. Basal body: tubulin *n* = 14, 182.9 ± 8.44; SAXO6-C *n* = 30, 181.8 ± 4.08; SAXO6-N *n* = 40, 180.6 ± 4.67. Connecting cilia: tubulin *n* = 14, 141.6 ± 11.28; SAXO6-C *n* = 29, 141.5 ± 4.05; SAXO6-N *n* = 40, 134.7 ± 3.81. (C) Stacked bar charts displaying the range of doublet microtubules on which SAXO6 was seen to localize in individual immuno-gold TEM cross-section images. *n* = cross-sections are indicated on each layer. Totals: *n* = 38 rod axonemes, *n* = 60 rod-connecting cilia, and *n* = 6 cone axonemes. (D) 3D representations of SAXO6 staining in rods and cones with iU-ExM, showing the number of continuous SAXO6 projections along the cilium.

To gain further insight into the spatial relationship between SAXO6 and microtubules in the whole photoreceptor cilia, transverse views of the photoreceptors were examined using iU-ExM and Ig-TEM. From a transverse plane, rods and cones can be differentiated based on the size difference of their ISs and centrioles (Figure S3A). Furthermore, different regions along the axial length of the photoreceptor cilium can be grossly identified based on the shape and arrangement of the MTDs, microtubule triplets, and presence or absence of accessory structures (i.e., distal appendages of the BB) (Figure 5A, TEM and schematics). When examining the localization of SAXO6 throughout the cilium, SAXO6 was found to co-localize with tubulin (Figure 5A). In line with what was previously shown,^11^^,^^42^ iU-ExM revealed SAXO6 localization throughout the length of the DCs in both rods and cones, while POC5’s presence extended partially through the photoreceptor DCs (Figure S3B).

Width measurements of the CC, BB, and DC by SAXO6 staining further showed that diameter values did not differ significantly from those measured by tubulin staining, though C-terminal SAXO6 staining was generally wider than tubulin, while N-terminal SAXO6 staining exhibited the opposite trend (Figure 5B). This contrasts with the observed width of a known CC inner scaffold protein, POC5, which was significantly reduced compared to tubulin staining (Figure S3C). SAXO6 was sometimes observed to be more luminal compared to tubulin-positive staining, but based on our width measurements, this pattern may be a consequence of the angle of the cilium relative to the imaging plane rather than a true differential localization. Ig-TEM also confirmed SAXO6 localization directly on microtubules within all ciliary regions observed (Figures 5A and S3D). Interestingly, through iU-ExM and Ig-TEM, SAXO6 was observed to localize to all nine MTDs in the BB; however, within the CC and axoneme, it was often co-localized with fewer MTDs (Figure 5A). This was particularly notable in the OS axoneme of rods, where most nanogold-labeled TEM cross-sections (50%) showed SAXO6 co-localization with 6–7 MTDs, exclusively not adjacent to the OS membranes (Figures 5C and 5D). In contrast, SAXO6 was observed to co-localize with all 9 MTDs in almost all of the observed nanogold-labeled TEM cross-sections from cone photoreceptors (Figures 5C and 5D). When visualizing the 3D z stacks of SAXO6 staining from expanded photoreceptors, it was also clear that SAXO6 localization was consistent along specific microtubules and did not seem to switch from one to another along the axial length of the cilium (Figure 5D; Video S2).

### SAXO6 co-localizes with individual ciliary MTDs in motile cilia

**Figure 6:**
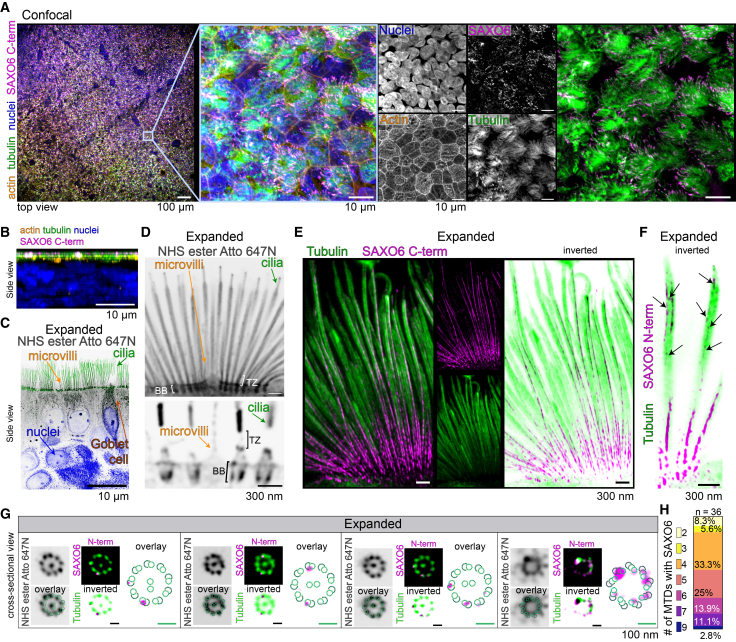
SAXO6 co-localizes with ciliary microtubules in motile cilia of a human iPSC-derived airway model (A and B) Confocal images of fully differentiated airway epithelium after 33 days of air exposure, stained for cilia (tubulin, green), microvilli (actin, orange), and SAXO6 (magenta) from either the top view (A) or side view (B). (C–F) Deconvolved confocal side-view images of expanded airway epithelium, stained for tubulin (green), NHS-ester (gray), and SAXO6 (magenta), with (C) and (D) showing the epithelial cell and ciliary architecture in this model. In (F), arrows point to SAXO6 labeling in the axonemes. (G) Deconvolved confocal images of individual cilia cross-sections from expanded cells, stained for tubulin (green), NHS-ester (gray), and SAXO6 (magenta), shown with a schematic overlay of the basal body or axoneme for reference. (H) Stacked bar charts displaying the range of doublet microtubules on which SAXO6 was seen to localize in iU-ExM cross-section images of individual axonemes from airway epithelial cells. *n* = 36 axonemes.

To establish if SAXO6 showed a similar distribution in motile cilia compared to immotile cilia of the photoreceptors, we used an airway epithelial induced pluripotent stem cell (iPSC)-derived model. The iPSC cells were grown on Transwells in an air-liquid interface, which allows for the establishment of all cell types and the organization found in human airway epithelia.^67^ Immunofluorescence with markers for filamentous actin (phalloidin) and motile cilia tufts (tubulin) was used to establish the localization of SAXO6 to the cilia in these cells (Figure 6A). From the side view, SAXO6 was observed in the apical region of the polarized epithelial cells, along with tubulin (Figure 6B). After undergoing iU-ExM, the cellular layers and types were even more visible, as shown with pan-staining with NHS-ester, where the cilia can be distinctly separated from the actin-filled microvilli or mucus-producing goblet cells (Figures 6C and 6D). SAXO6 was observed to co-localize with tubulin in these motile cilia, in the BB, and throughout the axoneme with both SAXO6 antibodies (Figure 6E). Intriguingly, SAXO6 staining appeared denser toward the base of the cilium, with lighter staining along the axial length, indicating that SAXO6 may be co-localizing with less than 9 MTDs in the axoneme (Figure 6F). This pattern of staining is consistent with the presence of MTDs, compared to singlets, as the B tubule is gradually lost in distal axonemes. Upon examination of the motile cilia cross-sections, the typical arrangement of 9 + 2 MTDs (central pair) was observed with NHS-ester and tubulin staining. SAXO6 was again observed co-localizing with ∼8–9 MTDs at the BB and base of the cilium, but in the axoneme, SAXO6 rarely co-localized with all 9 MTDs, with 58.3% of cross-sections revealing SAXO6 co-localization with 4–5 MTDs (Figures 6G and 6H).

### SAXO6 may be a MIP

**Figure 7:**
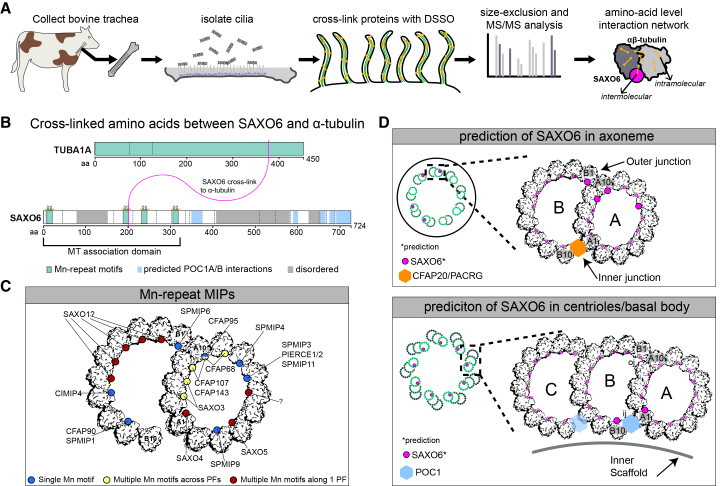
SAXO6 may localize to the ciliary microtubule inner lumen (A) Schematic of the workflow used for cross-linking mass spectrometry in bovine trachea. (B) Protein schematic of SAXO6 (GenBank: NP_001341898.1) including the Mn motifs (aa 9–21, 189–201, 232–244, and 306–318) and predicted POC1 interaction loci, and α-tubulin (GenBank: NP_006000.2) displaying the identified aa positions of their interaction (Lys370 on TUBA1A and Lys201 on SAXO6). (C) Microtubule doublet (structure taken from https://www.rcsb.org/structure/7RRO; PDB: 7RRO) with the localization of identified ciliary MIPs that also contain Mn motifs (modified from Leung et al.^81^). (D) Microtubule triplet or doublet with the mapped location of the SAXO6 cross-link on α-tubulin displayed in magenta. SAXO6 does not necessarily localize at every protofilament; therefore, predicted locations within the A or B tubules are indicated by larger magenta circles with ^∗^ (based on our data, similar motif MIPs or interactions with basal body protein POC1). Blue: POC1; orange: inner junction proteins CFAP20/PACRG; magenta: SAXO6.

Based on the nm-scale localization data described above, we hypothesized that SAXO6 is directly associated with ciliary microtubules. We therefore employed cross-linking (XL) MS (XL/MS) to identify tubulin-binding proteins from isolated cilia. This technique reveals interaction sites between (intermolecular) and within (intramolecular) proteins at the amino acid level (Figure 7A). To this end, we analyzed multi-ciliated cells from bovine trachea, a tissue from which thousands of cilia can be efficiently isolated (Figure 7A). The MS results from microtubule-enriched fractions exposed intermolecular XL between TUBA and SAXO6 (Figure 7B). Since the cross-linker (DSSO) is a fixed length and the cross-linked protein samples are filtered through a size-exclusion column, this suggests that the physical distance between SAXO6-Lys201 and TUBA1A-Lys370 is 30 Å or less. Importantly, the tubulin link on SAXO6 is within the region previously reported to be important for microtubule association.^40^ These originally defined microtubule-interacting sequences are termed Mn motifs, which have been shown to be important domains in ciliary MIPs, specifically in SAXO proteins and MAP6.^13^^,^^77^^,^^78^ Furthermore, other Mn motifs have been shown to bind to TUBA specifically in the loop between the S9 and S10 strands, exactly the same position as the SAXO6-TUBA cross-link we present here.^13^^,^^79^^,^^80^ Cryo-electron tomography studies have mapped these Mn-motif-containing MIPs to multiple sites along the inner lumen of both A and B MTDs of motile cilia (see Figure 7C for examples of Mn-motif MIP localization in bovine sperm flagella). When we mapped the amino acid XL positions between SAXO6 and TUBA on the bovine trachea αβ-tubulin model of MTDs (PDB: 7RRO), SAXO6 indeed showed a similar distribution to other Mn-motif-containing MIPs on the interior of the microtubule protofilaments (Figure 7D, opaque magenta dots). However, XL does not provide information on which tubulin protofilament SAXO6 binds, and therefore, hypotheses regarding the potential sites of interaction can only be made based on previous literature (Figure 7D, larger magenta dots), as discussed below.

SAXO6 is predicted to be highly unstructured, lacking conserved domains beyond its Mn motifs. Correspondingly, AlphaFold3 failed to generate high-confidence model predictions, and no intramolecular cross-links were detected by XL/MS, consistent with a disordered conformation. Despite this, at least one Mn motif within SAXO6 was conserved in products from all RNA isoforms, and it is thus possible that all *SAXO6*-derived protein forms can associate with microtubules.^77^ These results indicate that SAXO6 may reside within the MTD lumen and preferentially interact with specific MTDs in both motile and immotile cilia, hinting at clues to its ciliary function.

## Discussion

In this study, we identified bi-allelic genotypes comprising six different loss-of-function variants (V1–V6) in *SAXO6* in human subjects diagnosed with late-onset RP or CRD. In addition to retinal disease, all of these individuals displayed non-ocular phenotypes, such as hypertension, late-onset hearing loss, and obesity. These additional features are typical of early-onset syndromic ciliopathies such as Bardet-Biedl syndrome (BBS [MIM: 209900]) and Alström syndrome (ALMS [MIM: 203800]).^82^^,^^83^^,^^84^^,^^85^ However, in our cohort, it is likely that these extra-ocular signs, which are common to the general population at older ages,^86^^,^^87^ are unrelated to the presence of *SAXO6* variants. An exception could be T2DM, the only feature that was observed in the majority of subjects investigated (five out of six individuals with retinal disease). T2DM is also prevalent in the general population,^88^ at a rate of 21%. Statistically, however, the enrichment of T2DM cases in this study seems to be significant compared to the general population (*p* = 0.0012, by chi-squared test). Given the limited number of affected individuals tested and the presence of family members with T2DM but no retinal disease, the association of this phenotype, as well as any other non-ocular phenotypes, should be investigated by further clinical and molecular tests.

MDM1 (now SAXO6) was originally described as mouse double minute nuclear protein 1, along with MDM2, following the analysis of an extrachromosomal fragment of transformed murine 3T3 cells.^89^^,^^90^ The literature regarding the role of MDM2 in cancer, as an E3 ubiquitin ligase and oncoprotein, is extensive (reviewed in Zhu et al.^91^). However, MDM2 shares no homology with SAXO6. Although it is possible that SAXO6 could also be involved in cancer regulation/progression, given its role in centriole duplication suppression, its presence within the same extrachromosomal DNA fragment is probably linked to its proximity to MDM2 on the mouse genome rather than a role in oncogenesis. The endogenous localization of SAXO6 seems to be specifically in cilia (and not cytoplasmic microtubules or, despite the name, the nucleus), based on the tissues so far explored from our study and others.^40^^,^^45^

*SAXO6* is a well-conserved gene with metazoan lineages.^92^ In mammals, RNA expression of SAXO6 is observed in most tissues, with the highest values being displayed in the retina, brain, testis, and colon,^93^^,^^94^ with no apparent tissue-specific transcript isoform (dbGaP accession: phs000424.v10.p2 on June 11, 2025). Previous studies have shown that suppression of SAXO6 leads to an aberrant centriole duplication in cultured RPE1 cells^40^ and that knockout animals display photoreceptor degeneration.^44^^,^^45^ Given these data and the importance of the cilium in the preservation of normal photoreceptor homeostasis, the presence of pathogenic variants in *SAXO6* is coherent with the development of retinal disease in humans.

However, out of more than 500 genes currently associated with IRDs,^30^ only a handful are associated with pathologies as diverse as RP and CRD.^95^^,^^96^^,^^97^^,^^98^^,^^99^ This phenotype variability is in general caused by the type of variants (e.g., missense vs. loss of function) or their position along the gene’s transcript. For instance, in *RPGR* (MIM: 312610), variants at the distal end of open reading frame 15 (ORF15) cause CRD by affecting the glutamylation of the encoded protein, while variants preceding that distal region result in RP.^100^ Out of the 11 *SAXO6* isoforms, only 7 are expressed in the retina (Figure S2), according to long-read RNA sequencing data.^63^ V1 is the only variant that affects all 7 of these isoforms, including the two shortest isoforms (GenBank: NM_020128.4 and NM_001205029.3), and was identified in the only family from our series that displayed CRD. We can speculate that these short isoforms may be more relevant to cone physiology than to rod homeostasis, although no currently available data allow the testing of this hypothesis.

Using iU-ExM and Ig-TEM, we confirmed SAXO6 co-localization with tubulin in the axonemes of both human photoreceptors and lung epithelial motile cilia. XL/MS on isolated bovine tracheal cilia further revealed a direct interaction between the Mn-motif-containing region of SAXO6 and the inner lumen of folded TUBA. Together, these findings establish SAXO6 as a potential ciliary MIP and suggest its evolutionary conservation across motile and non-motile cilia. However, given that the affected individuals described in this study were not diagnosed with any of the classic disorders related to defects in motile cilia, it may be that the function and precise location of SAXO6 in photoreceptor cilia are unique and warrant further investigation.

The Mn motifs of SAXO6 share features with those of SAXO MIPs and MAP6 proteins,^77^^,^^78^^,^^81^^,^^101^^,^^102^^,^^103^^,^^104^^,^^105^ all of which stabilize microtubules in an Mn-dependent manner.^40^^,^^43^^,^^77^ These MAP6 and SAXO proteins were reported to bind to TUBA, specifically in the loop between the S9 and S10 strands.^13^^,^^79^^,^^80^ Our XL/MS data place the interaction between SAXO6 and TUBA directly within this loop region (Lys370). The spacing of the Mn motifs in SAXO6 (∼32 nm between the first two motifs) differs from other SAXO proteins (∼8 nm/40 aa),^43^^,^^81^ suggesting that SAXO6 may bridge multiple αβ-tubulin heterodimers longitudinally within a protofilament. Interestingly, the highest fluorescent intensity of SAXO6 localization in both photoreceptors and airway epithelial cilia was observed at the proximal end of cilia, which is similar to that of MAP6D1, an Mn-motif-containing protein required for B-tubule nucleation and microtubule stability.^77^ Given that MAP6D1 also localizes to glutamylated MTDs,^77^ SAXO6 may similarly associate with polyglutamylated MTDs, potentially contributing to B-tubule nucleation and doublet stability rather than singlet microtubule stabilization. This hypothesis aligns well with the location of SAXO6’s proposed ortholog, FAP363, to protofilaments A10–A11 near the outer junction and A-tubule seam/B-tubule nucleation site, as was also suggested in previous studies.^43^

Conversely, there is literature on other MAPs related to IRDs that would place SAXO6 at the inner junction (rather than the outer junction) of the MTD. Protein interaction mapping has identified POC1A and POC1B, two centriolar proteins, as potential SAXO6 partners.^42^ These centriolar scaffold proteins stabilize microtubules^106^ and are implicated in CRD.^107^^,^^108^^,^^109^^,^^110^ Loss of POC1A/B reduces SAXO6 localization in centrioles,^42^ suggesting an interdependent relationship. The inner junction protein CFAP20, also linked to IRDs,^111^ may share functional overlap with SAXO6. Although a direct interaction has not been demonstrated, SAXO6 could influence inner junction stability, potentially through indirect interaction or spatial proximity to CFAP20 and POC1 at the inner junction-inner scaffold interface. However, in photoreceptors, POC1B localizes to the BB and distal centriole,^107^^,^^109^ whereas SAXO6 extends into the CC and OS, indicating distinct yet potentially complementary functions. Thus, SAXO6-associated retinal degeneration may result from mechanisms separate from inner scaffold destabilization. Lastly, CFAP20 has been described in multiple species for many years as a well-established inner junction protein,^15^^,^^16^^,^^112^^,^^113^ whereas SAXO6 has not been linked to ciliary structure up to now, indicating that CFAP20 may have a more distinctive role in motile and non-motile cilia, while the function of SAXO6 may be more essential in primary cilia, such as the photoreceptors. Further structural and biochemical analyses are needed to delineate SAXO6’s precise location within the MTDs and its contribution to ciliary assembly and microtubule stability.

In summary, our findings reveal *SAXO6* as a new gene associated with hereditary retinal dystrophies. We also identify SAXO6 as a component of the microtubule inner lumen, localized throughout the axonemes of motile and non-motile cilia. The retinal phenotypes observed in the subjects of this study and the localization of SAXO6 in cilia indicate that *SAXO6* variants are associated with a first-order ciliopathy. Furthermore, our results suggest that a relatively underexplored class of proteins in photoreceptors, MIPs, may play a crucial role in maintaining microtubule stability and cellular homeostasis and that their dysfunction may lead to vision loss.

## Data and code availability

All variants identified in this study have been deposited in the ClinVar database (https://www.ncbi.nlm.nih.gov/clinvar/). The XL network datasets are available on ProteomeXchange/PRIDE (https://www.proteomexchange.org/), under accession numbers MassIVE: MSV000100624 and ProteomeXchange: PXD073717.

## Acknowledgments

This work was supported by the 10.13039/501100001711Swiss National Science Foundation (grant #176097 to C.R. and grant #224900 to C.L.M.) and by the Swiss RetinAward to M.Q.; by the 10.13039/501100003243Ministry of Health of the Czech Republic (grants UNCE/24/MED/022, SVV 2600631, and NW24-06-00083 to P.L., L.D., M.V., and B.K.); and by the 10.13039/501100003977Israel Science Foundation (grant #331/24 to T.B.-Y.). S.L. was funded by an MRC Clinician Scientist Fellowship (UKRI440). S.R. was supported by the Foundation Fighting Blindness Career Development Award (CD-GE-0621-0809-RAD), a Radboudumc Starter Grant (OZI-23.009), and NWO Aspasia (015.021.028). We thank the lab of Urs Jenal (Biozentrum, University of Basel), specifically Nicole Thürkauf, for the gift of the lung epithelial cells used in this study. We thank the Imaging Core facility (IMCF, Biozentrum, University of Basel), particularly Alexia Loynton-Ferrand, for technical assistance provided on the Stellaris 8 Falcon microscope. We thank Danilo Ritz and the Proteomics Core facility at the Biozentrum, University of Basel. The authors would like to thank all patients and their families for their participation in this study.

## Author contributions

A.R.M., M.Q., and C.R. designed the study. A.R.M. and C.L.M. generated wet-lab experimental data. A.R.M. and A.P.M. collected human retinal tissue. M.Q. and C.L.M. were responsible for computer-assisted analyses. A.R.M., S.L., J.H.H., L.D., K.R., P.L., T.B.-Y., and M.Q. were involved in the genetic data generation and analysis. V.S., Z.Z.N., D. Zur, M.V., B.K., S.R., O.A.M., G.A., A.R.W., T.B.-Y., P.L., and D. Zobor contributed to the collection and evaluation of clinical data. B.D.E. and C.R. were responsible for project supervision. B.D.E., A.G.-M., and C.R. were responsible for the resources for all wet-lab experiments. A.R.M. wrote the original draft, and all authors reviewed the manuscript, notably C.R., M.Q., and D. Zobor. All authors approved the manuscript.

## Declaration of interests

The authors declare no competing interests.

## Web resources

ClinVar, https://www.ncbi.nlm.nih.gov/clinvar
dbGaP, https://dbgap.ncbi.nlm.nih.gov/home/
GenBank, https://www.ncbi.nlm.nih.gov/genbank/
gnomAD, https://gnomad.broadinstitute.org
Human Protein Atlas, https://proteinatlas.org
MassIVE, https://massive.ucsd.edu/ProteoSAFe/
MutScore, https://mutscore-wgt7hvakhq-ew.a.run.app/
ProteomeXchange, http://www.proteomexchange.org
RefSeq, https://ftp.ncbi.nlm.nih.gov/refseq
SpliceAI, https://spliceailookup.broadinstitute.org/
UCSC, http://genome.ucsc.edu/cgi-bin/hgTables

## Supplemental information

Document S1. Figures S1–S3

Table S1. Primers and antibodies used in this study

Table S2. Clinical assessment of affected individuals

Table S3. Variants of affected individuals

Document S2. Article plus supplemental information
