# Supplementary material for: Loss-of-function variants in SAXO6, encoding a microtubule inner protein of photoreceptor cilia, cause a late-onset retinal dystrophy
Source: Am J Hum Genet. 2026 Feb 24;113(3):582–99. doi: 10.1016/j.ajhg.2026.02.001 (PMC13087473; doi:10.1016/j.ajhg.2026.02.001)
Supplement: Document S1. Figures S1–S3 [file mmc1.pdf]

The American Journal of Human Genetics, Volume *113*

Supplemental information

Loss-of-function variants in *SAXO6*, encoding a microtubule inner protein of photoreceptor cilia, cause a late-onset retinal dystrophy

Abigail R. Moye, Caitlyn L. McCafferty, Siying Lin, Ji Hoon Han, Lubica Dudakova, Kim Rodenburg, Viktória Szabó, Zoltán Zsolt Nagy, Dinah Zur, Marie Vajter, Bohdan Kousal, Alexandre P. Moulin, Alexandra Graff-Meyer, Susanne Roosing, Omar A. Mahroo, Gavin Arno, Andrew R. Webster, Tamar Ben-Yosef, Petra Liskova, Benjamin D. Engel, Ditta Zobor, Mathieu Quinodoz, and Carlo Rivolta

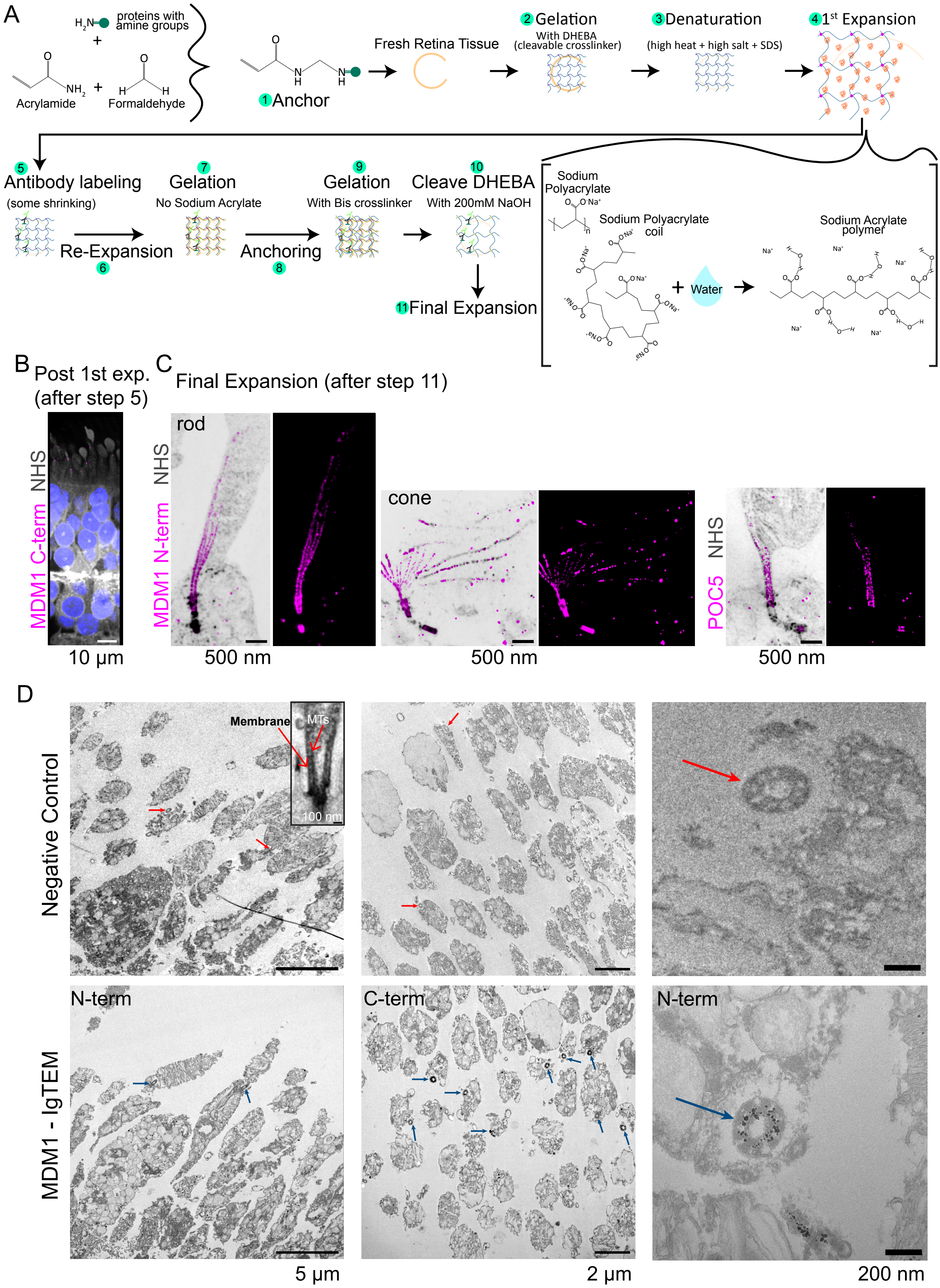

Figure S1. Expansion process and controls. (A) Schematics of the iterative ultrastructure expansion process used (see Methods). (B,C) Confocal images of human photoreceptor cilia after (B) the first expansion step indicated in panel (A), and (C) after the final expansion step. NHS-ester was used as a general cellular marker. DAPI stains nuclei (blue). Scale bars in (C) are corrected for a 10x expansion. (D) TEM micrographs of human photoreceptors showing negative control images (no primary antibody, but tissue labelled with nanogold, followed by silver enhancement) with no gold labelling or artefacts from silver enhancement, particularly in the cilium (marked with red arrows). Below, retina stained with MDM1 immunolabelling (blue arrows).

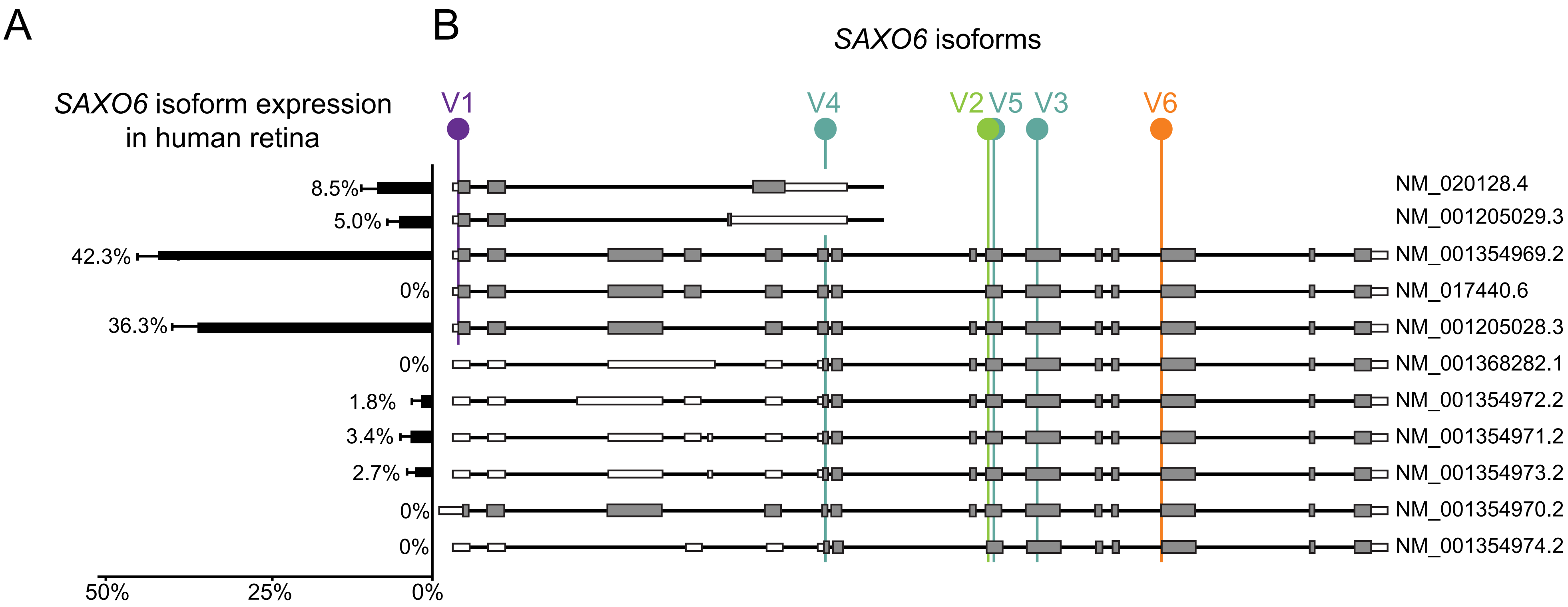

Figure S2. Isoforms affected by *SAXO6* variants. (A) Percentages of expression of the different *SAXO6* isoforms according to long-read RNA sequencing data from three human retina samples, showing 7 isoforms that are expressed in the retina. Data is represented as mean values with standard deviation. (B) Schematic diagram of all 11 *SAXO6* isoforms, showing which isoforms would be affected by variants V1-V6. **V1**: c.2T>C p.(Met1?); **V2**: c.1038del p.(Glu348AsnfsTer23); **V3**: c.1240A>T p.(Lys414Ter); **V4**: c.868A>T p.(Lys290Ter); **V5**: c.1048C>T p.(Arg350Ter); **V6**: c.1750-1G>C p.? .

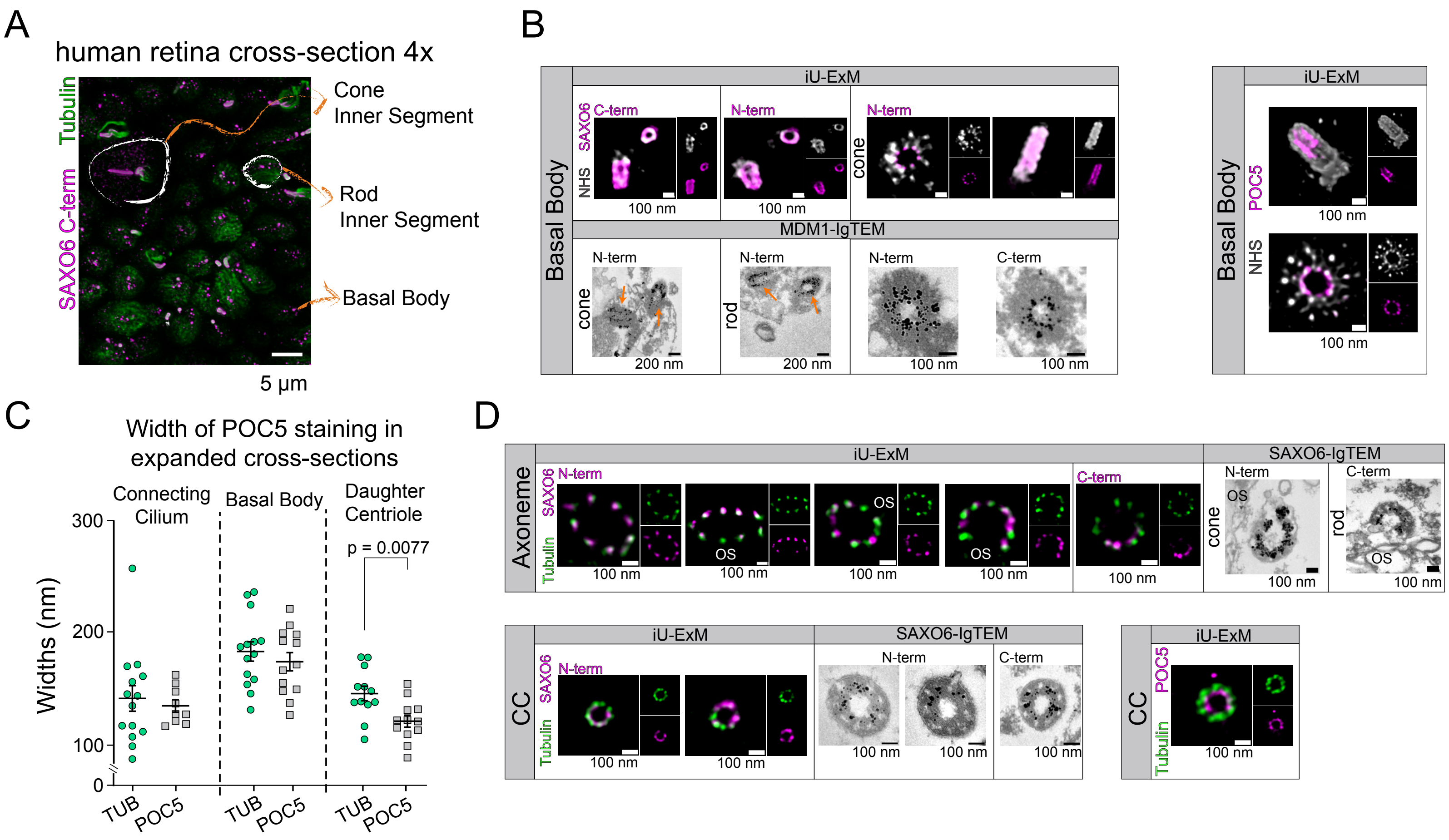

Figure S3. SAXO6 localization differs from inner scaffold protein POC5 in human retina. (A) Confocal deconvolved image of retina (post 1st expansion = gelled control), flipped 90º for viewing in the transverse plane. Photoreceptor inner segments are outlined. (B) Deconvolved confocal images of individual cilia from expanded retina or electron micrographs of human retina immunogold labeled for SAXO6, showing staining in the basal body and centrioles of either SAXO6 (magenta) or POC5 (magenta) with NHS (gray, used for basal body structure indications). (C) Scatter plot displaying widths of tubulin (used in Figure 5) and POC5 labelling from individual expanded cilia in deconvolved confocal images, in either daughter centrioles, basal bodies, or connecting cilia. Displayed as mean±SEM, and p values from unpaired t-tests with Welch's correction, showing a trend for decreased widths of POC5 labelling compared to tubulin (p-values are not shown for non-significant differences). Data were generated from four experiments, over 2 biological samples. Daughter centriole: tubulin n = 12, 146 ± 6.42 nm; POC5 n = 12, 121.6 ± 5.23. Basal body: tubulin n = 14, 182.9 ± 8.44, POC5 n = 13, 173.9 ± 8.0. Connecting cilium: tubulin n = 14, 141.6 ± 11.28, POC5 n = 9, 135.1 ± 5.47. (D) Deconvolved confocal images of individual cilia from expanded retina or electron micrographs of human retina immunogold labeled for SAXO6, showing staining in the connecting cilium (CC) or axoneme of either SAXO6 (magenta) or POC5 (magenta) with tubulin (green).
